# Multidetector Computer Tomography: Evaluation of Blunt Chest Trauma in Adults

**DOI:** 10.1155/2014/864369

**Published:** 2014-09-08

**Authors:** João Palas, António P. Matos, Vasco Mascarenhas, Vasco Herédia, Miguel Ramalho

**Affiliations:** ^1^Department of Radiology, Hospital Garcia de Orta, Avenida Torrado da Silva, Almada, 2801-951 Setúbal, Portugal; ^2^Department of Radiology, Hospital da Luz, Avenida Lusíada 100, 1500-650 Lisbon, Portugal; ^3^Department of Radiology, Hospital Espírito Santo, Largo do Senhor da Pobreza, 7000-811 Évora, Portugal

## Abstract

Imaging plays an essential part of chest trauma care. By definition, the employed imaging technique in the emergency setting should reach the correct diagnosis as fast as possible. In severe chest blunt trauma, multidetector computer tomography (MDCT) has become part of the initial workup, mainly due to its high sensitivity and diagnostic accuracy of the technique for the detection and characterization of thoracic injuries and also due to its wide availability in tertiary care centers. The aim of this paper is to review and illustrate a spectrum of characteristic MDCT findings of blunt traumatic injuries of the chest including the lungs, mediastinum, pleural space, and chest wall.

## 1. Introduction

In the United States and Western Europe, trauma is the fourth most common cause of death and the leading cause of death in the population with less than 45 years of age [1]. Thoracic injuries and related complications in the patient who has experienced blunt chest trauma have a mortality of 15.5% to 25% [2]. Traffic accidents are the major source of blunt chest trauma representing approximately two thirds of the cases [3].

Imaging studies play an essential part of thoracic trauma care. The information generated with different diagnostic imaging tools has a major role in management of chest trauma patients [4]. The ideal imaging technique should reach the correct diagnosis as fast as possible. Chest radiography (CXR) has been the traditional screening technique to evaluate thoracic trauma. However, the information obtained is suboptimal for the diagnosis of vascular and nonvascular thoracic injuries, as it underestimates the severity and extent of chest trauma and, in some cases, fails to detect the presence of injury [5]. There is growing evidence that multidetector computed tomography (MDCT) is more sensitive than CXR in the detection and characterization of thoracic injuries after trauma [6, 7]. Traub et al. found that 42% patients had additional findings reported by MDCT scan beyond that found on their CXR [6]. Brink et al. [8] found additional findings in up to 59% of patients with MDCT compared with CXR and Trupka et al. reported clinical changes in management after CT scans in up to 70% of patients [9].

Due to its wide availability, speed, and ability to depict a variety of injuries, as well as being able to simultaneously evaluate other body regions (e.g., abdomen and pelvis), MDCT is now considered the gold standard imaging tool in the emergency department [4], particularly in trauma centers (level 1) and larger hospitals that have CT technologists and radiologists available 24 hours per day [7].

Optimal assessment requires careful technique, including the use of intravenous iodinated contrast media. MDCT's increasingly faster acquisition times as well as the significantly improved spatial resolution allow angiography, multiplanar reconstructions, and volume rendering techniques for clinical application. In severely injured patients and unstable patients, CXR remains the most used diagnostic modality, but it seems that in hemodynamically stable patients applying MDCT scan as the first-line diagnostic modality in blunt chest trauma can accelerate diagnosis as well as treatment, reduce costs, and result in better outcome [5]. Due to its high accuracy, and recent developments on dose reduction, MDCT is now being increasingly used in less severely injured trauma population.

Drawbacks of MDCT include the radiation exposure and the potential adverse effects related with the use of contrast media. Additionally, MDCT is also associated with higher costs and increasing time spent in the emergency department [10]. It is necessary to weigh a risk-benefit analysis taking into account the type of trauma (high energy versus low energy), patient's age, clinical parameters, and expected follow-up exams.

Most algorithms subsequently recommend selective MDCT; there is increasing evidence that, instead of selective MDCT of the chest, a routine thoracic and abdominal MDCT might be preferable as it reveals more injuries, with higher injury severity scores [1]. It should be noted that there is no clinical predictors that can rule out all important traumatic injuries and there is no clear evidence in which situation CT can be safely omitted without missing relevant injuries [8].

This paper reviews and illustrates a spectrum of characteristic MDCT findings of traumatic chest injuries.

## 2. CT Protocols

According to the type of MDCT available, a collimation of 1.25 mm (4-slice and 16-slice) or 0.6 mm (64-slice) is recommended. Usually we use a tube voltage of 120 kVp and a variable tube current time product (mAs), as Automatic Exposure Control (AEC) is regularly applied.

Our protocol includes injection of intravenous administration of contrast medium, many times only acquiring postcontrast imaging, so as not to miss any injury of the major mediastinal vessels and the heart. Optimal opacification may be obtained with injection of 100–120 mL of iodinated contrast medium at a flow rate of 3-4 mL/s and a delay of 25–40 s. If MDCT of the thorax is part of a whole body trauma CT, then a compromise can be made with a 60 s delay for the whole body.

When active bleeding is suspected, a delayed acquisition at 4-5 min is recommended, provided that the patient is hemodynamically stable [11, 12]. Due to time constraints we do not recommend the systematic use of ECG gating for chest trauma [13, 14]. Our standardized protocol includes reconstruction in a soft tissue, lung and bone windows. The axial thin slice acquisition can be used to create multiplanar reconstructions and volume-rendered images.

## 3. Lung Parenchymal Trauma

### 3.1. Pulmonary Contusion

**Figure 1:**
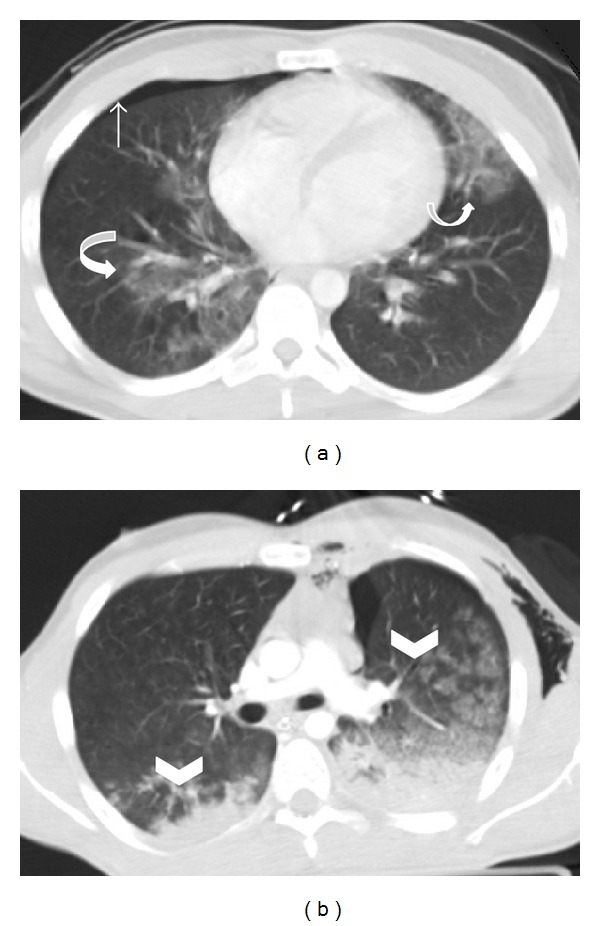
Bilateral pulmonary contusion. Axial MDCT in lung window reveals (a) ill-defined nonsegmental areas of “ground glass” attenuation in middle lobe, right inferior lobe, and lingula in a polytraumatized patient, consistent with bilateral contusion focus (curved arrows). Also note a small right pneumothorax (straight arrow). Axial MDCT of another patient (b) shows “ground glass” lung contusions (arrowheads) and bilateral nonsegmental air space consolidations with a posterior distribution due to blood filling of the alveolar spaces.

Pulmonary contusion is the most common pulmonary lesion and is seen in 30%–70% of patients with blunt chest trauma [7, 15]. It is a focal parenchymal injury of the alveolar epithelium, with interstitial edema and alveolar hemorrhage, produced at the time of injury and usually adjacent to the area of trauma, but can also occur in a countercoup location [1, 3]. MDCT is precise in the diagnosis and quantification of the extent of pulmonary contusions. The appearance of pulmonary contusions depends on the severity of the parenchymal injury. In mild contusion, ill defined, patchy, “ground-glass” areas of heterogeneous opacities are generally seen and are related with interstitial or partial alveolar compromise (Figure 1). When alveolar injury is moderate to severe, it is seen as poorly defined areas of consolidation, with no air bronchogram sign, as a result of bronchial obstruction caused by secretions and/or blood. Massive pulmonary contusion may lead to the development of adult respiratory distress syndrome [7].

**Figure 2:**
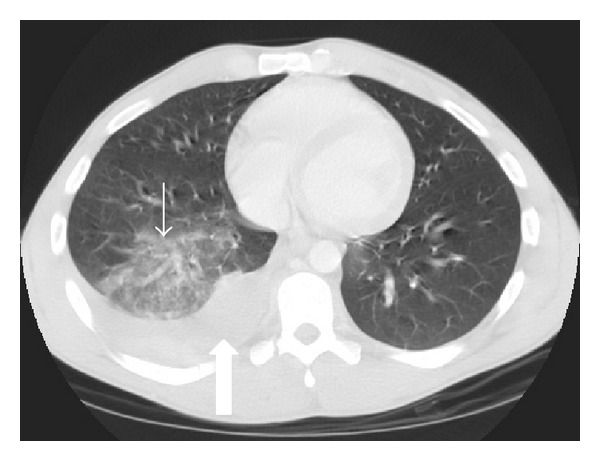
Pulmonary contusion and hemothorax in a patient who fell down of his bike. Axial MDCT in lung window at the level of left cardiac chamber shows right lower lung contusion (thin arrow) associated with ipsilateral hemothorax (thick arrow).

Pulmonary contusions may be associated with other lesions, such as chest wall contusions or fractures in the overlying area of impact, hemothorax (Figure 2), pneumothorax, or lacerations.

Resolution is usually rapid and the lung often returns to normal within a week. Failure of resolution usually suggests superimposed infection, atelectasis, aspiration, or a blood clot in a laceration.

### 3.2. Lung Laceration

**Figure 3:**
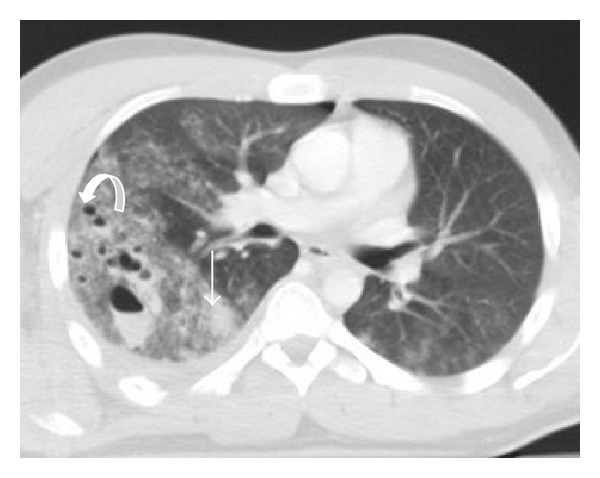
Pulmonary lacerations. Axial MDCT in lung window at the level of pulmonary trunk. Multiple focus of pulmonary lacerations can be depicted, some of them are filled with air (pneumatocele, curved arrow), others filled with blood (hematocele-straight arrow), and some filled with both, making an air-liquid level (pneumo-hematocele, arrowhead). Surrounding pulmonary contusions are appreciated. Associated left pulmonary contusions and a small right pneumothorax are also depicted.

Lung laceration refers to a traumatic disruption of alveolar spaces with cavity formation filled with blood (hematoma), air (pneumatocele), or, more frequently, a combination of both (hemato-pneumatocele) (Figure 3) [7, 15]. Lacerations are commonly solitary, but multiple lacerations may occur. Laceration was previously considered a rare finding. Nowadays, due to the broad use of MDCT, lacerations appear relatively common in blunt chest trauma [16]. Blunt chest trauma can produce substantial pulmonary lacerations; nevertheless they are most commonly caused by penetrating traumas such as stab or bullet wounds [17].

MDCT is superior to CXR to detect lacerations. The laceration may be lucent and filled with air, completely opacified as a result of blood accumulation within the cavity, or demonstrate an air-fluid level related to variable amounts of blood within its lumen [18]. The resultant pneumatocele has a variable course; it may persist for several weeks, although it usually resolves within one to three weeks, resulting in a pulmonary parenchymal scar [17].

Conservative treatment is the rule as most of these lesions usually resolve within weeks. Surgery is commonly indicated in cases of large parenchymal destruction, bleeding from a major vessel, or bronchovascular fistula [19].

## 4. Mediastinal Trauma

### 4.1. Pneumomediastinum

Pneumomediastinum is defined as free air collections surrounding mediastinal structures and dissecting along the mediastinal fat. Both overt and occult pneumomediastinum may occur in the setting of blunt chest trauma [20]. The presence of pneumomediastinum should raise suspicion for a tracheobronchial or esophageal rupture. Frequently it originates in an alveolar rupture. It fills the interstitium and then reaches the hilum and mediastinum, dissecting along the bronchovascular sheaths (Macklin effect) [3, 21]. If under pressure, air in the mediastinum might produce cardiovascular disturbance, which may be fatal if not treated immediately [17]. Pneumomediastinum may be mistaken for pneumothorax, but the presence of septa within it, delineated on lung window, helps in differentiating the two findings, especially if they coexist [3].

### 4.2. Tracheobronchial Laceration

Tracheobronchial injuries occur in less than 1.5% of blunt chest trauma patients. Bronchial tear is more common than tracheal tear and more often on the right side. Approximately 85% of tracheal lacerations occur 2 cm above the carina and are usually located at the cartilage-membranous junction [7]. Blunt trauma may cause an abrupt increase in intrathoracic airways pressure. If this happens against a closed glottis, a tracheobronchial laceration may occur [18, 22].

**Figure 4:**
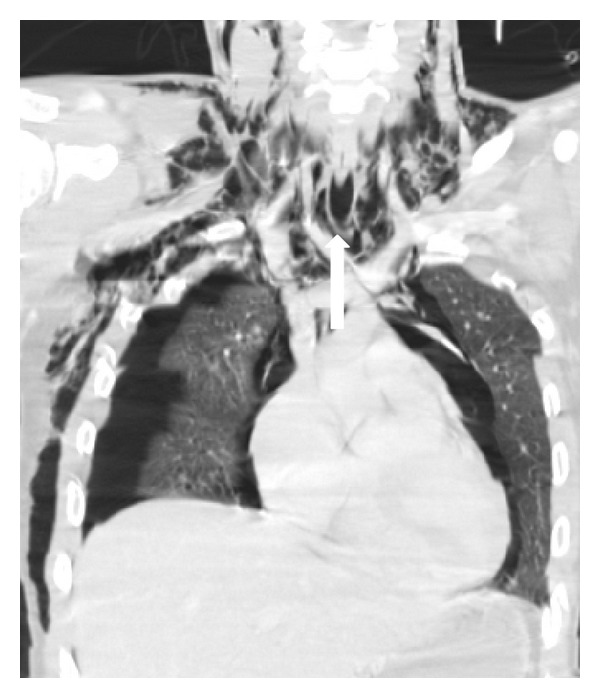
Tracheal rupture. Coronal reconstruction of axial MDCT in lung window. An extensive subcutaneous emphysema, bilateral pneumothorax, and pneumomediastinum are observed. Close attention to the tracheal wall depicted a small leak of air to the mediastinum (arrow).

Discontinuity of the tracheal or bronchial wall may be seen, although infrequently, with air leaking around the airway (Figure 4). Other less specific signs of tracheobronchial tear include collapsed lung (“fallen lung” sign), persistent pneumothorax, and herniation or over distention of an endotracheal cuff in an intubated patient [7, 23, 24]. MDCT is also very effective in evaluating central airway permeability.

Repair of tracheobronchial lacerations should be performed promptly due to its high mortality rate and to avoid chronic pulmonary complications [18].

### 4.3. Esophageal Injury

Blunt trauma of the esophagus is rare, due to its position in the mediastinum. Usually it is secondary to violent vomiting (Boerhaave's syndrome) or compressive bone forces [25]. The cervical esophagus has been reported as the most common site of injury. When esophageal rupture occurs, it is a nearly fatal condition and the associated mortality approaches 90% (almost always secondary to mediastinitis [17]).

MDCT findings that might suggest traumatic esophageal perforation include the presence of pneumomediastinum, mediastinitis, hydropneumothorax, or leakage of oral contrast material into the mediastinum or pleural space [26].

### 4.4. Large Chest Vessels Lesions

The aorta is the most commonly affected vessel. Rarely, injuries of the aortic branches, pulmonary arteries, internal thoracic artery, or major mediastinal veins may occur [16]. Blunt traumatic aortic injury is associated with significant mortality. It was historically estimated that over 75% of patients experienced prehospital mortality, and of those arriving to the hospital alive, up to 50% died within the first 24 hours following injury [27, 28]. Contemporary data suggest that approximately 4% of patients die during transport to the hospital and that 20% of these patients die early in their hospital course [29].

A blunt trauma can damage the thoracic aorta by several mechanisms (e.g., fracture dislocating thoracic vertebras; penetration of the first rib and clavicle), but the majority occurs after significant decelerating traumas, so that some authors believe that all victims of decelerating traumas, such as motor vehicle crashes, should be referred to an angiographic examination of the aorta [30].

The currently accepted grading system for these injuries was proposed in 2009 [31] and has been adopted by the Society for Vascular Surgery (SVS) in the clinical practice guidelines for management of thoracic blunt traumatic aortic injury [32]. In this grading system, injuries are assigned to one of four categories: grade I (intimal tear); grade II (intramural hematoma); grade III (pseudoaneurysm); and grade IV (rupture). Current guidelines from the SVS recommend endovascular repair of grade II–IV injuries of the thoracic aorta [32]. A recent investigation by Osgood et al. showed that injury progression in grade I-II is rare (≈5%) and did not cause death in their study cohort, proposing imaging follow-up for grade II [33]. The traumatic lesions of the thoracic aorta typically occur in the aortic isthmus, aortic arch, and descending aorta at the level of diaphragm [25]. Contrast enhanced MDCT of the chest has been promoted as an effective screening tool.

**Figure 5:**
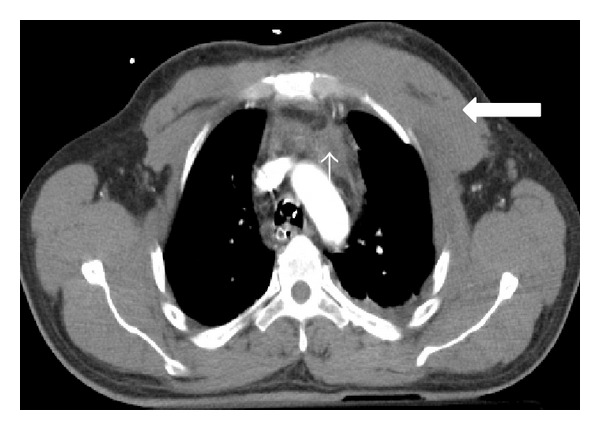
Polytraumatized patient who was hit by a car. Axial MDCT after intravenous contrast administration, at the level of aortic arch, demonstrates mediastinal hemorrhage (thin arrow) and left anterior chest muscle wall hematoma (pectoralis major, thick arrow).

**Figure 6:**
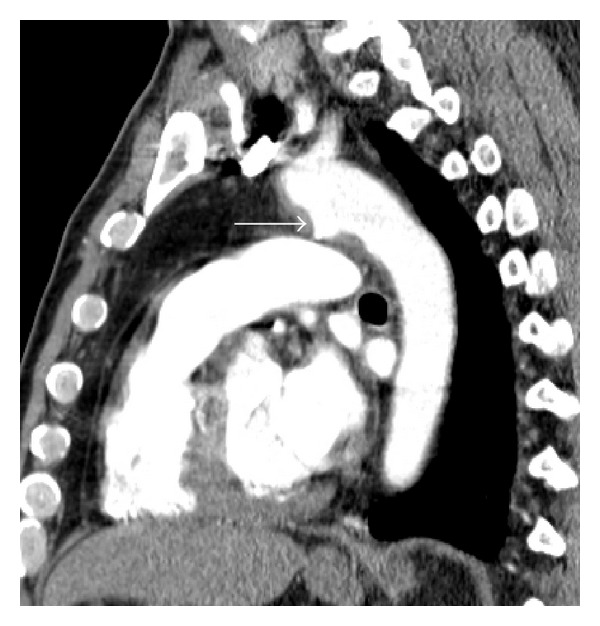
Thoracic aortic pseudoaneurysm in the context of blunt chest trauma. Sagittal reconstruction of arterial phase MDCT demonstrates an abnormal contour of the thoracic aorta. A sacculation filled with iodinated contrast material involving the anterior aspect of transition of the aortic arch with the descending aorta, immediately after the emergency of the left subclavian artery, consistent with aortic pseudoaneurysm (arrow).

Findings associated with aortic lesions include mediastinal hemorrhage (Figure 5), aortic-contour deformity, intimal flap, intramural hematoma, direct evidence of a tear, thrombus into the aortic lumen, pseudoaneurysm (Figure 6), abrupt tapering of the descending aorta relative to the ascending aorta (“pseudocoarctation”), and rupture with extravasation of contrast material [22, 25]. Injuries to the supra-aortic and pulmonary arteries and large venous vessels (vena cava, azygos) may be associated with cardiac tamponade or hypovolemic shock from massive hemorrhage [25].

### 4.5. Hemopericardium

**Figure 7:**
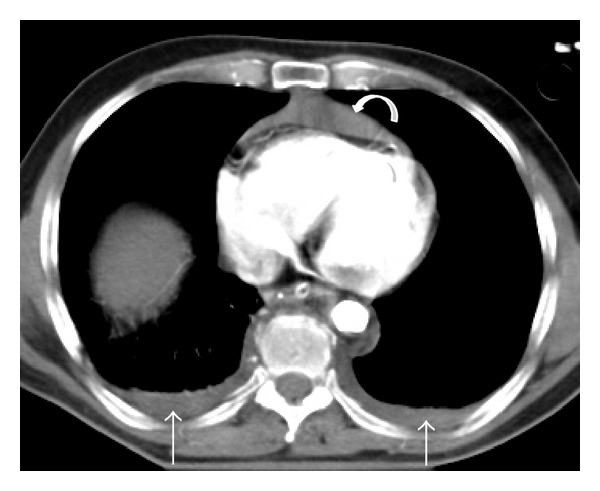
Hemopericardium and bilateral hemothorax. Postcontrast axial MDCT of a polytraumatized patient reveals a pericardial (curved arrow) and a bilateral pleural effusion (straight arrows), with high attenuation consistent with fresh blood content.

Hemopericardium is a rare condition in the setting of blunt chest trauma, usually caused by venous hemorrhage but may also be caused by cardiac injury or secondary to ascending aorta rupture. MDCT can detect hemopericardium before the onset of pericardial tamponade [23]. MDCT findings include pericardial blood effusion (Figure 7), with or without dilation of the superior and inferior vena cava. Other findings include reflux of contrast material into the azygos vein and inferior vena cava, deformation and compression of cardiac chambers and other intrapericardial structures, and bulging of the interventricular septum [34].

### 4.6. Pneumopericardium

Esophageal ruptures or pleuropericardial fistulas may initiate air into the pericardial cavity [25]. It is a very rare finding, but if large, it may result in cardiac tamponade [16].

**Figure 8:**
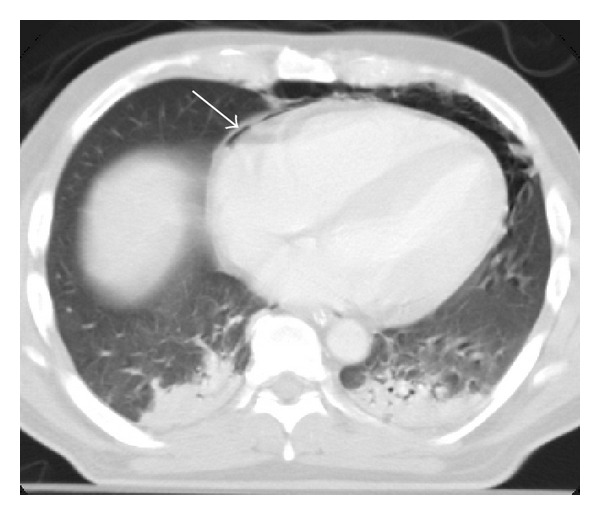
Pneumopericardium. A thin line of air is appreciated between the pericardium layers (arrow). Bilateral parenchymal contusions were also present.

Findings include air around the heart that does not rise above the level of pericardial reflection at the root of the great vessels (Figure 8).

## 5. Injuries of the Pleural Space

### 5.1. Hemothorax

Hemothorax is defined as a collection of blood in the pleural space, usually due to lesions of the lung parenchyma, pleura, chest wall, mediastinum, or abdomen (liver and splenic injuries with diaphragmatic rupture). It occurs in 30%–50% of patients who suffer blunt chest trauma [7].

**Figure 9:**
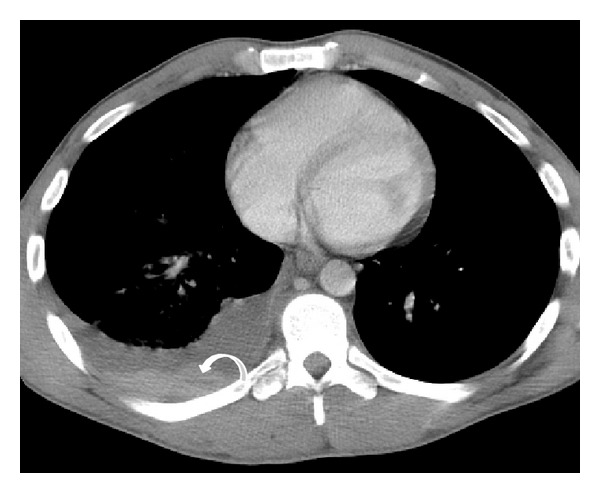
Right hemothorax with the “hematocrit sign.” Postcontrast axial MDCT at the level of ventricular chambers demonstrates a right pleural effusion with a liquid-liquid level (curved arrow), giving an aspect of layered effusion, consistent with right hemothorax with different degrees of blood coagulation (“hematocrit sign”).

**Figure 10:**
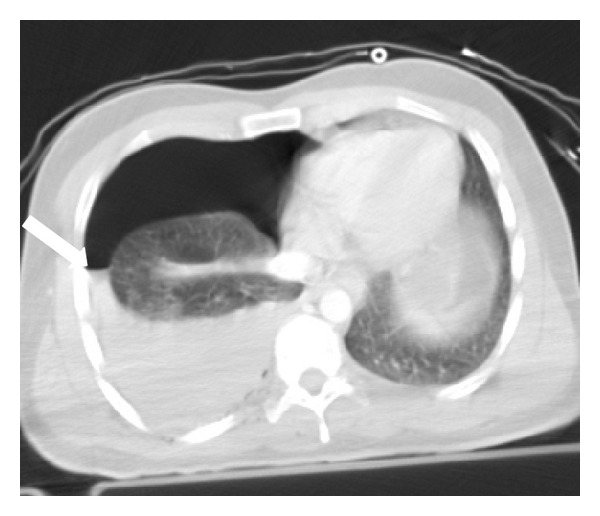
Right hemothorax and pneumothorax. Postcontrast axial MCDT shows a right hemopneumothorax creating an air-liquid level (arrow).

MDCT easily characterizes the pleural fluid and determines the value of attenuation (typically presents with an attenuation of 35–70 H.U.) [26]. Blood can be seen in the pleural space at different degrees of coagulation, giving rise to a layered appearance, called the “hematocrit sign” (Figure 9). MDCT is also more sensitive than CXR in detecting small hemothoraces [2]. The combination of pneumothorax and hemothorax is common (Figure 10) [19].

### 5.2. Pneumothorax

Pneumothorax occurs in 30–39% of cases of blunt chest trauma [35–37]. It represents an abnormal collection of air in the pleural space between the visceral and parietal pleura. Mechanisms include broken alveoli due to sudden increase in the intrathoracic pressure, chest deceleration (with or without rib fractures), ruptured emphysematous bulla, pulmonary laceration, or tracheobronchial injury or due to the “Macklin effect” [38].

MDCT has higher sensitivity than CXR in the detection of pneumothorax, particularly in the supine trauma patient [25, 39]. Pneumothoraces that are not apparent on the supine chest radiograph have been shown on CT in 10% to 50% of patients [25]. The detection of small volume pneumothorax has clinical importance, since artificial ventilation may worsen this condition.

**Figure 11:**
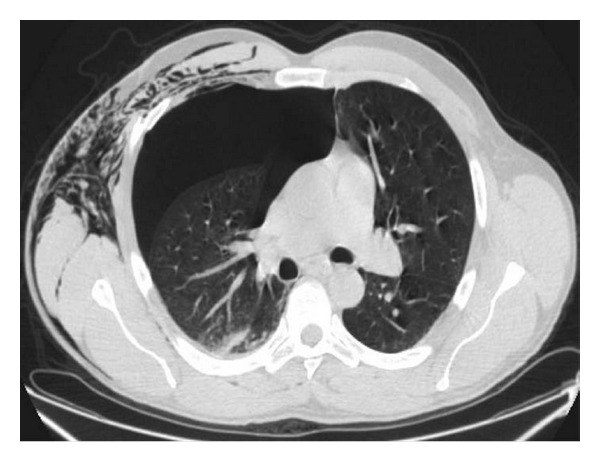
Tension pneumothorax. Axial MDCT in lung window at the level of the pulmonary trunk shows increased volume of the right hemithorax due to a large pneumothorax. This finding reduces the ipsilateral pulmonary volume and shifts the mediastinum to the left. A small contusion focus in the posterior segment of the right upper lobe and subcutaneous emphysema are also seen. This is an indication for immediate chest drainage.

Tension pneumothorax develops when air enters the pleural space but cannot leave and progressively accumulates as a result of a one-way valve mechanism. It expands the ipsilateral hemithorax, collapses the associated lung, depresses the associated hemidiaphragm, displaces the mediastinum to the opposite side, produces atelectasis in the contralateral lung, and prevents adequate diastolic filling of the heart, by compressing of the vena cava. These imaging features might be depicted with MDCT (Figure 11). The cardiorespiratory distress caused by tension pneumothorax might be severe [17].

The treatment of choice is pleural drainage. If hemodynamic impairment is suspected, prompt decompression with a thoracostomy tube while the patient is in the CT suite is possible [1], as well as immediate replacement of eventual malpositioned chest tubes [39]. Surgery is usually indicated when there is a persistent or massive air leak or lack of lung reexpansion [19].

## 6. Chest Wall and Diaphragm Trauma

### 6.1. Rib Fractures

**Figure 12:**
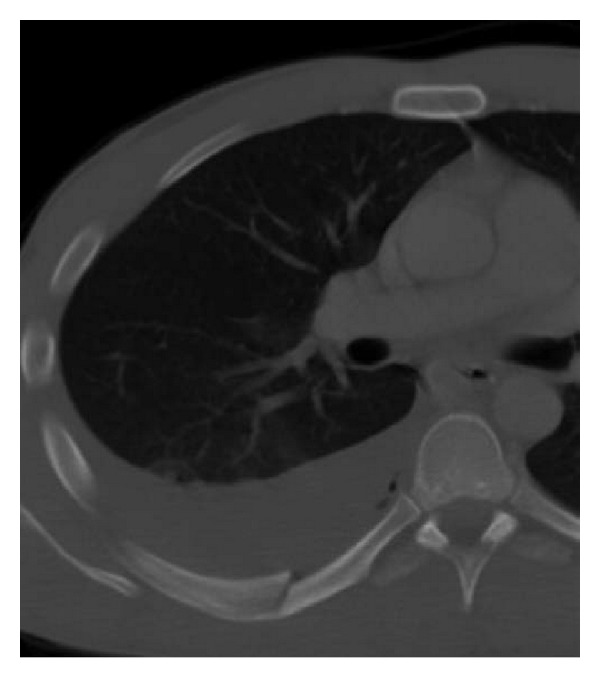
Rib fracture. Axial MDCT in bone window at the level of pulmonary trunk clearly demonstrates a fracture bone line of 8th posterior right arch associated with ipsilateral hemothorax.

Rib fractures are the most common lesion occurring in the setting of blunt chest trauma. They are usually identified on MDCT scans obtained following blunt chest trauma, being observed in 50% of patients (Figure 12) [3]. The fourth to the eighth arches are the most commonly affected ribs [3]. Fractures involving the first through the third ribs are a marker of high-energy trauma, as they are mostly protected by the clavicle, scapula, and upper chest wall musculature. Injury to the brachial plexus and subclavian vessels may be seen in 3% to 15% of patients who have upper rib fractures [40]. Fractures of the eighth to eleventh ribs should prompt careful evaluation for upper abdominal organ injuries [7, 26].

**Figure 13:**
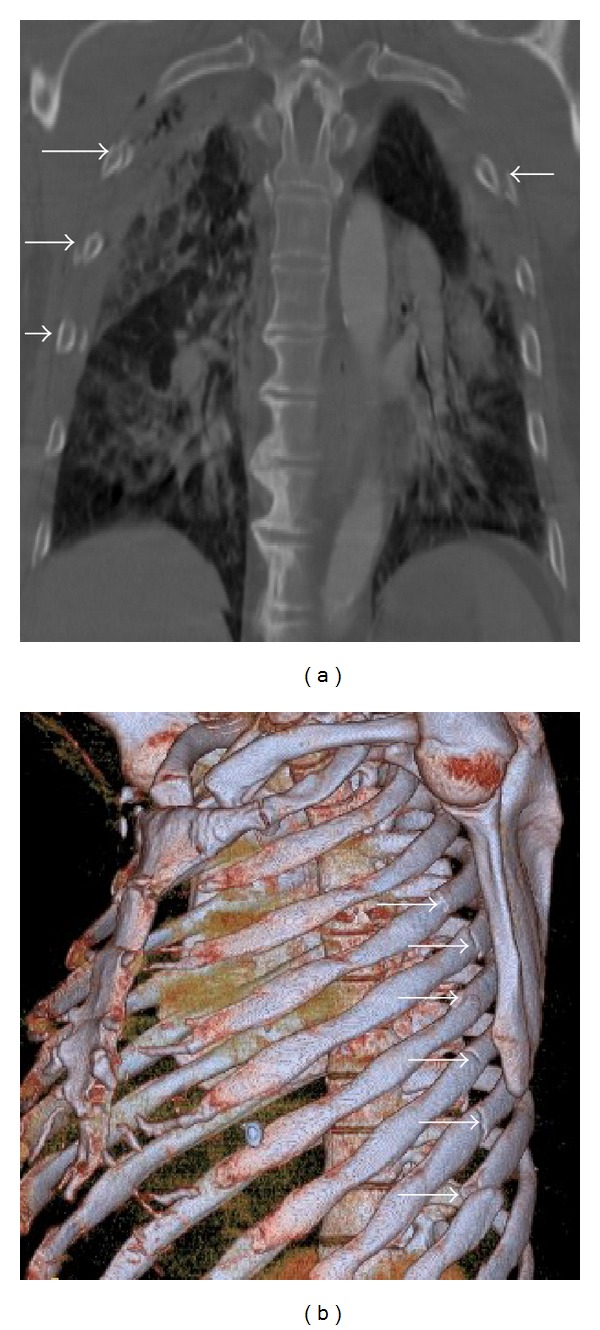
Rib fractures in 2 polytraumatized patients. Coronal MDCT in bone window (a) in a case of “flail chest” with four displaced rib fractures (straight arrows) in three consecutive right costal arches and in one left costal arc. Note the associated pulmonary contusions. Another patient (b) presenting with multiple left rib fractures (arrows), shown with oblique sagittal volume-rendering reconstruction.

Flail chest is a traumatic condition in which there are three or more consecutive ribs with fractures in two or more places, often requiring surgical treatment (Figure 13) [7, 16].

Many of these rib fractures are not shown on the initial CXR. MDCT can determine the site and number of fractures, as well as other associated injuries (hemothorax, pneumothorax, subcutaneous emphysema, and pulmonary contusion) [23].

Treatment should be aimed to maintain a good respiratory function and control the pain. If required, mechanical ventilation for pneumatic stabilization of the chest can be performed. Adequate results have been reported with noninvasive mechanical ventilation in CPAP (Continuous Positive Airway Pressure) mode [19, 41]. Chest surgical stabilization is only indicated when the patient requires a thoracotomy for other reasons or has massive flail chest that might not be solved with mechanical ventilation [19].

### 6.2. Sternal Fractures

**Figure 14:**
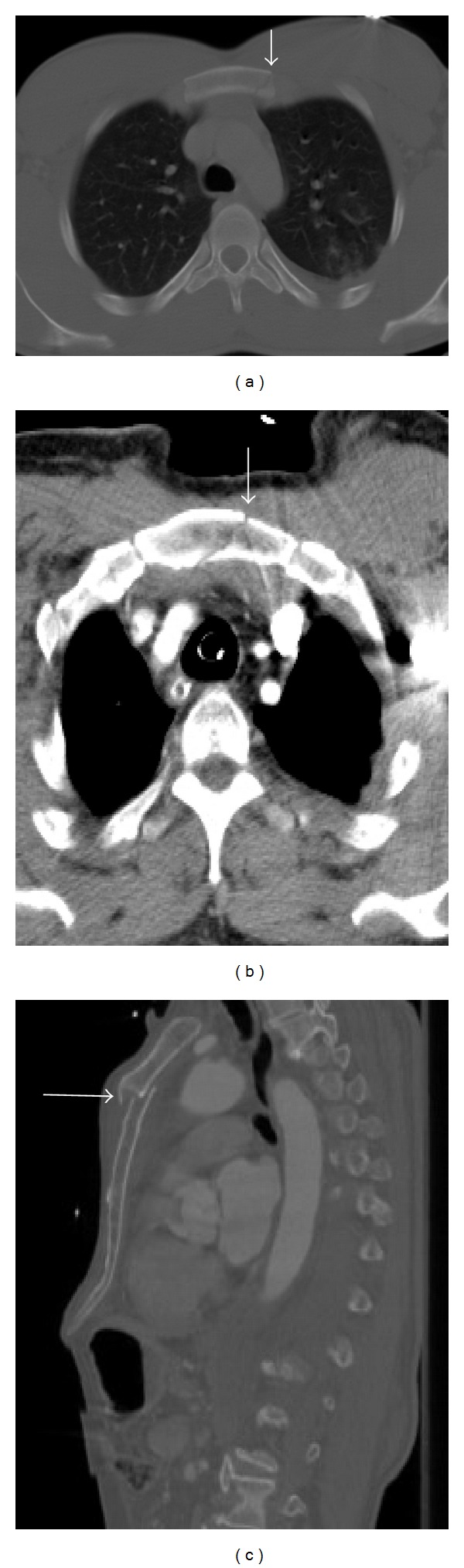
Sternum fracture in two different patients. Axial MDCT (bone window) in patient one (a) shows a complete sternum fracture at the level of the body, without displacement of the fragments (arrow). Axial MDCT (b) and sagittal reconstruction in bone window (c) in a second patient show a displaced sternal body fracture (arrows). A small retrosternal hematoma is also seen (b).

Sternal fractures have been reported in approximately 8% of blunt chest trauma patients [42]. Approximately 90% of such fractures are secondary to motor vehicle accident (due to seat belt or air bag trauma) [7]. They usually involve the sternal body and manubrium (Figure 14) and are often associated with mediastinal hematoma, lung lesions, and cardiac or spinal injuries. If vascular compromise or impingement is a concern, intravenous contrast should be administered.

The fracture is usually obvious at MDCT, often with an associated retrosternal mediastinal hematoma [15]. Multiplanar and three-dimensional reconstructions greatly improve accuracy and diagnostic confidence. In this setting, sagittal images are particularly helpful for the detection of sternal fractures; however, stair-step artifacts of the sternum may be seen on sagittal reformations due to respiration. Another common pitfall is the presence of constitutional abnormalities of the sternum segmentation, mimicking sternal fractures. Treatment is usually based on pain control and chest physical therapy [19].

### 6.3. Clavicle Fractures

**Figure 15:**
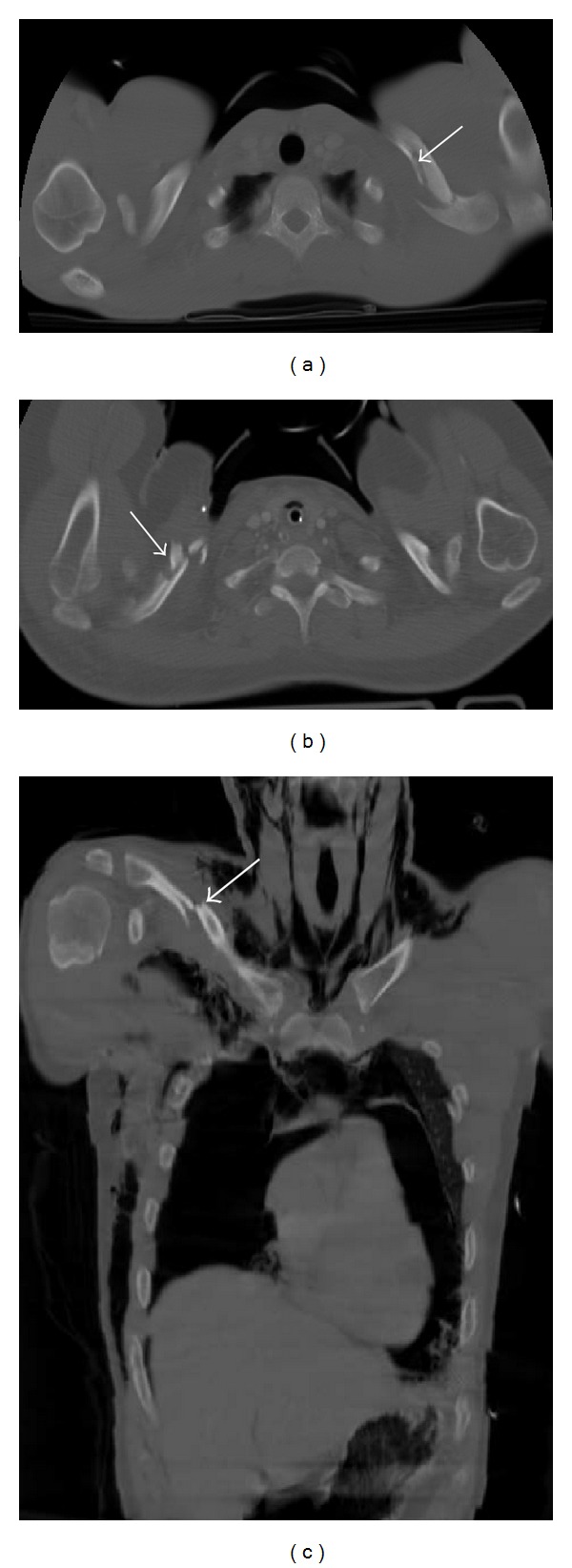
Three cases of clavicle fractures. Axial MDCT reconstruction in bone window ((a), (b)) and coronal reconstruction in bone window (c). A two-fracture line is seen in the left clavicle (a); a comminuted fracture is seen in the right clavicle, with multiple fragments (b); and a middle third fracture with dislocation is seen in the right clavicle (c). In (c) there are associated left anterior costal arc fracture, right pneumothorax, pneumomediastinum, and subcutaneous emphysema.

Clavicle fractures are usually obvious on the clinical examination. The most important role of MDCT in clavicle fracture evaluation relies in the assessment of medial fractures and injuries affecting the sternoclavicular joint, especially in the diagnosis of sternoclavicular dislocation [16]. Anterior sternoclavicular dislocation is more common and it is a marker for high-energy trauma as patients usually have other chest injuries (Figure 15). A posterior sternoclavicular dislocation may be a cause of serious morbidity, but it is often clinically and radiographically occult, only being detected on chest CT [7]. Impingement of the underlying mediastinal vessels and nerves, such as the brachial plexus and recurrent laryngeal nerve, esophagus, and trachea, can occur by the displaced clavicle [2, 3]. If vascular compromise or impingement is a concern, the study should be performed with intravenous contrast enhancement. Treatment often requires open reduction [2].

### 6.4. Scapular Fracture

**Figure 16:**
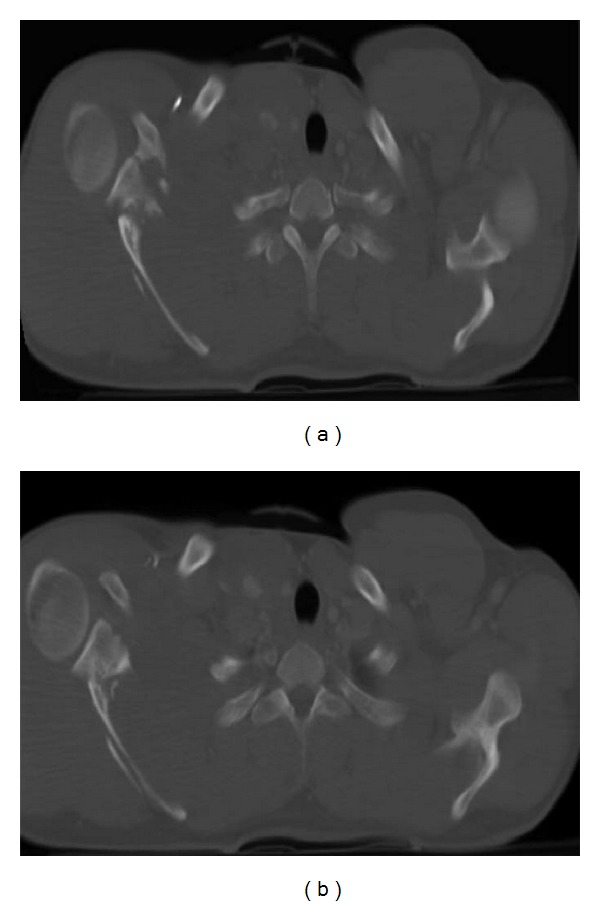
Right scapular fracture. Axial MDCT in bone window ((a), (b)). Comminuted right scapular fracture involving the scapular neck and spine is clearly observed.

Scapular fractures are relatively common. Usually it is necessary a significant force for it to occur because the scapula is protected by the large muscle masses of the posterior thorax (Figure 16) [2]. Although most scapular fractures are treated nonoperatively, any fracture involving the glenoid or scapular neck requires open reduction and internal fixation to allow normal scapulothoracic motion and stabilization of the shoulder girdle [2]. They are often associated with pulmonary contusion, rib, clavicle, and vertebral fractures and arterial injuries (subclavian, axillary, or brachial) [3]. These injuries are usually well seen in MDCT and multiplanar reconstructions are helpful.

### 6.5. Thoracic Spine Fractures

Fractures of the thoracic spine occur in 3% of patients with blunt thoracic trauma [43]; a high percentage is associated with spinal cord injury. The most common site in this setting is the thoracoabdominal junction at the level of T9–T11 vertebral bodies [15].

**Figure 17:**
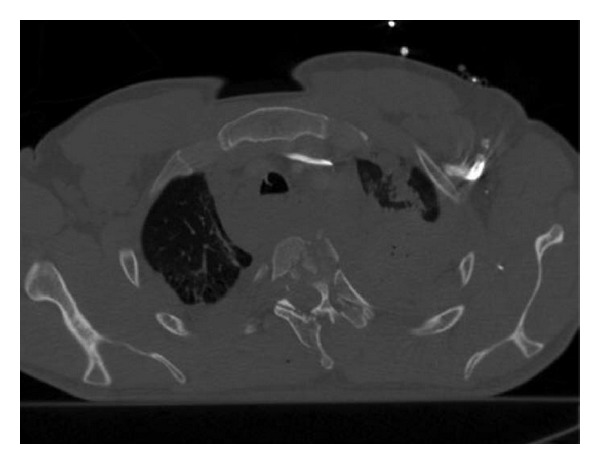
Thoracic vertebral fracture in a patient who suffered a car crash. Axial MDCT in bone window. A comminuted thoracic vertebral fracture is depicted with multiple fragments of the body and spinous processes of the third thoracic vertebra. Hemomediastinum, hemothorax, and pulmonary contusions are also associated.

MDCT is the modality of choice in the evaluation of spinal fractures. Signs of vertebral body fractures include disruption or fracture of the vertebral body, pedicle, and/or spinous processes, paraspinal hematoma, and confined posterior mediastinum hematoma (Figure 17) [22].

MDCT shows the presence and extent of a spinal injury, predicts the degree of instability produced, and can show bony fragments in the neural canal. Reconstructed sagittal and coronal multiplanar images are often useful [15, 23].

### 6.6. Chest Wall Hematoma

Chest wall hematoma is a relatively infrequent complication of a chest wall injury or of dedicated chest interventions, including drainage or insertion of a central venous catheter [16].

Hematomas may be of arterial or venous origin (Figure 5). Extrapleural hematomas are commonly associated with rib fractures that injure the intercostal, internal mammary, or subclavian arteries [7]. Blood accumulates between the parietal pleura and endothoracic fascia. Larger hematomas have a biconvex shape. Active bleeding may be seen [16].

### 6.7. Subcutaneous Emphysema

**Figure 18:**
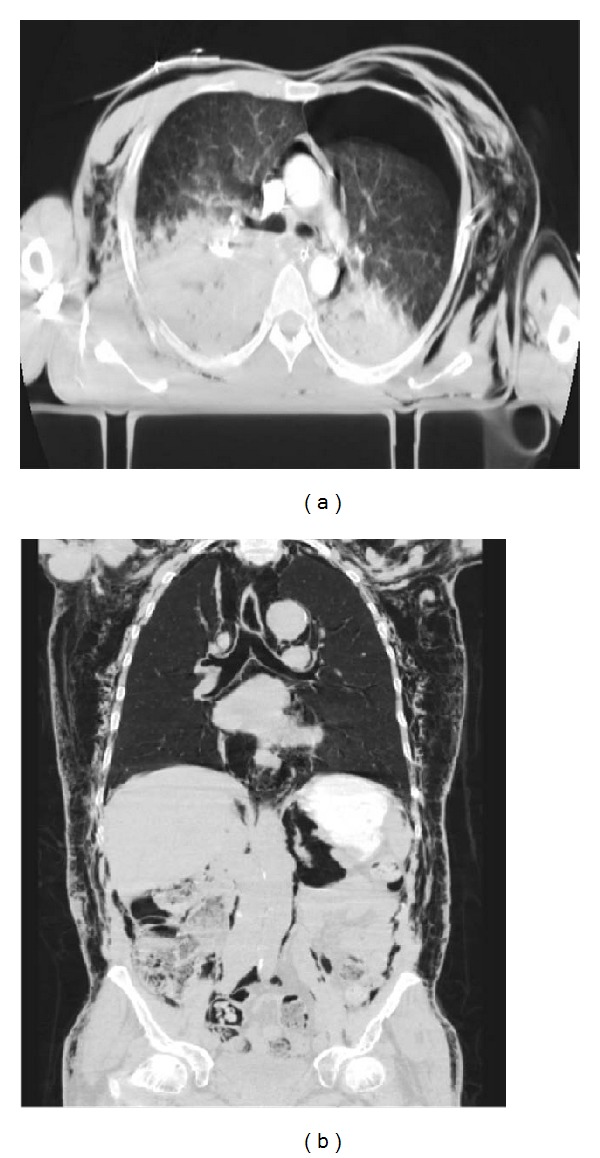
Subcutaneous emphysema. Axial MDCT (a) and coronal reconstruction (b) in lung window. An extensive subcutaneous emphysema is observed. A pneumomediastinum and retropneumoperitoneum are also associated.

Air can spread through the fascial planes to the remainder of the chest wall, abdomen, or even into the head, neck, and extremities (Figure 18) [25]. Most of the times it has a tracheobronchial tear origin, but it can also be a consequence of esophageal rupture.

### 6.8. Lung Herniation

Lung herniation is a rare complication of blunt chest trauma where pleural-covered lung extrudes through a defect in the thoracic wall [7]. It occurs at the site of an inherited or acquired defect of the chest wall with a significant increase in intrathoracic pressure. The acquired chest wall defects can be caused by multiple fractures of the ribs or by sterno- and costoclavicular dislocation [3].

The diagnosis is commonly achieved with MDCT, demonstrating the extent of chest wall injury and the amount of herniated lung. The anterolateral chest wall is more susceptible to traumatic lung herniation, because of the minimal soft tissue support (intercostal muscles) compared to the posterior wall. Supraclavicular hernias have also been reported. Treatment is required when lung herniation is symptomatic, usually by surgical reduction [7].

### 6.9. Diaphragmatic Trauma

Diaphragmatic rupture occurs in 0.8–7% of patients hospitalized with a blunt trauma [44]. It is a frequently overlooked injury but it is clinically very serious. Mechanisms of diaphragmatic rupture after blunt trauma include a sudden increase in intrathoracic or intra-abdominal pressure while the diaphragm is immovable by a crushing force [18].

**Figure 19:**
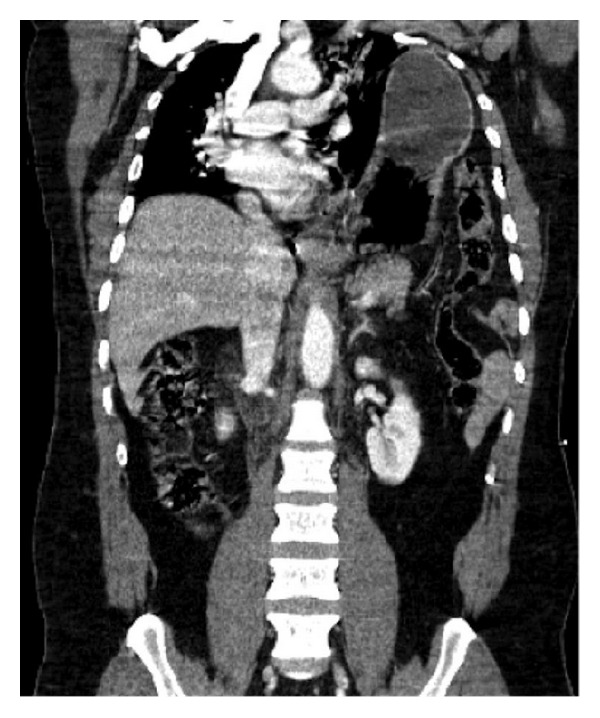
Signs of rupture of the diaphragm. Coronal MDCT reconstruction. A massive left diaphragmatic hernia with herniation of the stomach and left colon content is seen in a patient who suffered a car accident. It decreases left pulmonary volume and shifts the mediastinum towards right.

MDCT not only detects small diaphragmatic discontinuities, but also identifies the herniated fat or viscera. Usually there is a waist like constriction of the herniated stomach or bowel (collar sign) or lack of visualization of the hemidiaphragm [18, 25]. Coronal and sagittal reformations are essential in detecting diaphragmatic rupture (Figure 19).

Most diaphragmatic ruptures originate in the posterolateral portion of the diaphragm at the site of embryonic diaphragmatic fusion [3] and it is more common on the left side (77–90%), presumably because the liver protects the right hemidiaphragm [18]. Notably, the stomach is the most common herniated abdominal organ.

False-positive interpretations are usually due to the loss of continuity of the diaphragm seen in older patients with incidental Bochdalek hernias [15].

Surgical repair is necessary to prevent late complications such as bowel incarceration or strangulation, thoracic organ compression, and diaphragmatic paralysis [25].

## 7. Conclusion

This paper reviewed a broad spectrum of characteristic MDCT findings of traumatic chest injuries. Although conventional radiography plays an important role in the initial emergency room setting in patients with chest trauma, MDCT has clearly established itself as the principal imaging method for this patient group, owing to its wide availability, rapid access, quick implementation, use of standardized protocols, and the possibility of generating multiplanar and three-dimensional reconstructions. The information provided by MDCT may lead to critical changes in patients' management; thus we believe that clinicians, radiologists, and radiology residents should be familiar with the different aspects of MDCT evaluation of this subset of patients.
